# AIM: AI for microbiology

**DOI:** 10.1093/nsr/nwaf225

**Published:** 2025-06-25

**Authors:** Jun Wang, George Fu Gao

**Affiliations:** CAS Key Laboratory of Pathogen Microbiology and Immunology, Institute of Microbiology, Chinese Academy of Sciences, China; CAS Key Laboratory of Pathogen Microbiology and Immunology, Institute of Microbiology, Chinese Academy of Sciences, China

The 21st century awaits further development in biomedical research and technology to address the challenges in climate change, food and agriculture innovation, supply chain resilience and human health in the coming decades. Such bold goals should largely be made possible due to the developments in artificial intelligence (AI) that have been made in the past decade or so, namely the fourth wave of science and technology (S&T) revolution or the third wave of AI as we are currently experiencing. In the eminently influential book *The Structure of Scientific Revolutions*, the American philosopher Thomas Kuhn named the non-linear, periodical revolutions and accelerations in science ‘paradigm shifts’, which are usually the result of technological and methodological improvements which then contribute disproportionately more than the linear and gradual accumulation of scientific discoveries and knowledge. Nobel Laureate Sydney Brenner also said ‘progress in science depends on new techniques, new discoveries and new ideas, probably in that order’. Since the beginning of the 20th century, the fields of biology and medicine have undergone several paradigm shifts that have tremendously sped up their development, including but not limited to the discovery of DNA as genetic material and the central dogma, revelation of host immune system (antibody discovery and T cell receptor cloning), Sanger sequencing and polymerase chain reactions (PCR), identification of the CRISPR-Cas and consequent development into gene-editing tools, and more recently second- and third-generation sequencing.

None of the discoveries or developments, however, would have been possible without microbiology, a relatively young field that only began in the 1860s with the emergence of pivotal frameworks including germ theories and Koch's postulates; and in turn, the identification of 16S rRNA as phylogenetic markers by Carl Woese in the 1970s and application of next-generation sequencing (NGS) that gave rise to the molecular taxonomy of three domains of life (including bacteria and archaea, besides eukaryotes) and the division of microbiome research, respectively. Last but not the least, recombinant DNA technology and discovery of microbial enzymes for gene modifications, enable modern drug developments. Now it's time for AI, the newest workhorse to combine with the wagon of microbiology, and bring forth the newest generation of productivities [1]. Here, we predict the new paradigm in microbiology research of AI in microbiology (AIM) to be ‘generative microbiology’, which will give precise ‘aims’ for novel drug discoveries, making longevity (say 120 years old) a reality.

The most important definition of such generative microbiology should be the deliverables, the lack of which was exactly the most important reason for the failure of the last two waves of AI which eventually dissipated into two AI ‘winters’. Defining a new direction or paradigm for microbiology requires the exact delineation of the expected outcome, rather than the methodological requirements [2]. Generative microbiology would deliver: artificial and novel genes for the development of research tools and clinical treatments, as well as industrial production; universal vaccines for infections, metabolic and immunological diseases, neurodegenerative diseases and cancer; and lastly, *de novo* designed proteins and macromolecular machinery, even microbial species and communities. In this Special Topic, we gathered experts from this field to present their own methodology, findings and views as an AIM issue; and a comprehensive review on state-of-art methods is first provided by Karin and Steinegger; bioinformatics is probably the earliest bracer of AI advances. A subbranch of machine learning, deep learning encompasses the state-of-art neural network approaches underlying most of the recent developments. The authors present a comprehensive list of tools available for metagenomic research, including the merits and caveats of each, and recommend the ‘best practice’ for using those tools for microbiology research [3].

From a genetic perspective, the extant genes and metabolic pathways from various microbes have already been widely studied and utilized in production of small metabolites and macromolecules for medicinal and industrial applications; they also provide the expanding set of tools for biomedical research including gene editing systems. Peng and Fu offer their perspective on the utility of bioinformatic tools in identifying key mobile elements in the gut microbiome, showcasing their analytical power and new insights [4]. Such mobile elements greatly expand the genetic and coding diversity of the gut microbiome, moving beyond a static picture of metagenomics; such genetic variations were however difficult to detect in previous studies due to technical and bioinformatic constraints. As a special section, Steinegger also gives an interview on his take of new AI approaches, and what it will mean for biology in the following decades [5]. As a main contributor to ground-breaking tools including MMseqs2, FoldSeek and ColabFold, he recounts his academic training and engagement in AI research. The changes brought by AI such as AlphaFold and other methods are ‘revolutionizing biology’, he commented. Assisted by AI, we expect to learn from the accumulated knowledge and generate new genes and gene sets that greatly improve on the ones currently available in a significantly reduced time, and achieve completely novel functions that are not present in natural organisms.

At the same time, microbiome research in the last decade has revealed the contribution of complex microbiomes to the development of metabolic, immunological and neurodegenerative diseases as well as cancer, and once these causal roles are established, prophylactic vaccines are in theory feasible to prime the immune system to prevent the microbial triggers of various diseases, in a similar fashion to vaccines against infectious pathogens. By deep-learning of nanopore data, Cao and colleagues reveal that methylation signatures in the gut microbiome can be used to distinguish disease at single-species level, and more importantly such epigenetic modifications can be manipulated to increase phage therapy efficacy [6]. From this additional layer of data, they discovered that such epigenetic signals are species-, individual- and disease-specific, associated with gene expression, and even phage therapies could benefit from modulating epigenetic levels of the host-phage interaction system. Moreover, learning from the accumulated knowledge of pathogen-associated molecular patterns (PAMPs), bacterial and viral effector proteins, generative AI is expected to provide a solution for universal vaccines against influenza or coronaviruses, even capable of preventing immune escapes. Ma and colleagues address the ever-evolving threat from SARS-CoV-2 and the ongoing development of countermeasures, empowered and also enabled by AI developments [7]. Empowered with the highest number of genomes available for a single species of virus, we now have a large amount of information on the mutational landscape of the SARS-CoV-2 genome, yet predicting their evolutionary trajectory and pathogenicity, as well as immune evasion remain challenging. Liu on the other hand offers his insights on the utility of improving nutritional studies and applications with deep-learning [8]. The concept of individualized or precise nutrition has been proposed for decades, but only with the accumulation of individual microbiome response to dietary interventions and integration by deep learning approaches, will such goals become feasible. The ultimate goal would be a vaccine combination, generated towards microbial and host components, that can delay gaining and expanding the lifespan.

Last, after successful generation of separate genes or proteins, the next step would be generation of protein and macromolecular machinery, and eventually microbial species and communities. Protein complexes can be generated by design, or even de novo design, to function in a similar modes or improved fashion to extant cellular components or organisms. For example, an artificially designed protein machinery can mimic the function of bacteriophages and treat antibiotic-resistant pathogens, a threat that is becoming more challenging, while overcoming the current limitations of phage host range and biosafety concerns. A primer for this would be predicting protein-protein interactions with confidence, and one such tool is presented by Tao and colleagues in this issue [9]. Using deep learning tools, changes in binding affinity can be reliably predicted for engineered enzymes or monoclonal antibodies, facilitating the design and future generation of enzyme/antibody for industrial, environmental and medical applications. From there, we can envision artificially generated cellular organisms with the necessary genes for designated purposes, and logically mixed organisms to form communities (‘micro-cosmos’), to be used in humans, animals and plants, agriculture and food processing, and in the environment for health monitoring and maintenance.

Echoing Richard Feynman's last writing, ‘What I cannot create, I do not understand’, the AIM is probably the field that has already combined a balanced proportion of descriptive studies and applications, and the use of AI in microbiology is surely not behind the other subfields of biomedicine. With AI having already demonstrated its power in assisting in microbial taxonomy, metabolite identification and mining of functional peptides [10], generative microbiology eyes at the applicable future of AIM, and the deliverables are expected to greatly accelerate production of medicine and healthy food, reducing pollution and carbon emissions in industrial production, and revolutionize research in biomedicine.

***Conflict of interest statement.*** None declared.
